# Lower Insulin-Dose Adjusted A1c (IDAA1c) Is Associated With Less Complications in Individuals With Type 1 Diabetes Treated With Hematopoetic Stem-Cell Transplantation and Conventional Therapy

**DOI:** 10.3389/fendo.2019.00747

**Published:** 2019-11-19

**Authors:** Jaquellyne Gurgel Penaforte-Saboia, Carlos Eduardo Barra Couri, Virginia Oliveira Fernandes, Ana Paula Dias Rangel Montenegro, Lívia Aline De Araújo Batista, Lenita Zajdenverg, Carlos Antonio Negrato, Kelen Cristina Ribeiro Malmegrim, Daniela Aparecida Moraes, Juliana Bernardes Elias Dias, Maria Carolina Oliveira, Akhtar Hussain, Marilia Brito Gomes, Renan Magalhães Montenegro

**Affiliations:** ^1^Postgraduate Program in Medical Sciences, Department of Clinical Medicine, Federal University of Ceará, Fortaleza, Brazil; ^2^Clinical Research Unit, Walter Cantidio University Hospital, Federal University of Ceará/EBSERH, Fortaleza, Brazil; ^3^Center for Cell-Based Therapy, Regional Blood Center of Ribeirão Preto, Ribeirão Preto Medical School, University of São Paulo, Ribeirão Preto, Brazil; ^4^Department of Internal Medicine, Ribeirão Preto Medical School, University of São Paulo, Ribeirão Preto, Brazil; ^5^Postgraduate Program in Public Health, Department of Community Health, Federal University of Ceará, Fortaleza, Brazil; ^6^University Hospital, Federal University of Rio de Janeiro, Rio de Janeiro, Brazil; ^7^Brazilian Society of Diabetes, São Paulo, Brazil; ^8^Faculty of Health Sciences, Nord University, Bodø, Norway; ^9^Centre for Global Health Research, Diabetic Association of Bangladesh, Dhaka, Bangladesh; ^10^Diabetes Unit, Department of Internal Medicine, State University of Rio de Janeiro, Rio de Janeiro, Brazil

**Keywords:** type 1 diabetes (T1D), IDAA1c, residual B-cell function, glycemic control, microvascular complications

## Abstract

**Objective:** To evaluate the association between insulin-dose adjusted A1C (IDAA1c) and microvascular complications (MC) and hypoglycemia in a representative Brazilian population of Type 1 diabetes mellitus (T1DM) patients.

**Research Design and Methods:** This was a cross-sectional study based on a previous study, “Microvascular Complications in Type 1 Diabetes: a comparative analysis of patients treated with autologous nonmyeloablative hematopoietic stem-cell transplantation (AHST) and conventional medical therapy (CT)”. The 168 patients in that study (144 from CT plus 24 from AHST) were re-subdivided into two groups, according to their IDAA1c values (30 patients had IDAA1c ≤ 9; 138 had IDAA1c > 9). Then, the prevalence of MC (diabetic renal disease, neuropathy, and retinopathy), hypoglycemia (blood glucose <60 mg/dL), and severe hypoglycemic (episode of hypoglycemia that required the assistance of another person to treat) events were compared between the groups. The groups were well-matched on these factors: duration of disease, sex, and age at the time of diagnosis of T1DM.

**Results:** After an average of 8 years after diagnosis, only 6.6% (2/30) of the patients from IDAA1c ≤ 9 group developed any MC, whereas 21.0% (29/138) from the IDAA1c > 9 group had at least one complication (*p* = 0.044). Regarding hypoglycemic events, the proportion of individuals who reported at least 1 episode of hypoglycemia in the last month was 43.3 and 64.7% from the IDAA1c ≤ 9 and IDAA1c > 9 groups, respectively (*p* = 0.030). Regarding severe hypoglycemia, the proportion of patients presenting at least one episode in the last month and the rate of episode/patient/month were similar between groups (6.7 vs. 13.2%; *p* = 0.535; and 0.1/patient/month vs. 0.25/patient/month; *p* = 0.321).

**Conclusion:** In a representative Brazilian population of T1DM patients, those with IDAA1c ≤ 9 presented a lower frequency of MC, as well as fewer episodes of hypoglycemia, in the month prior to the analysis.

## Introduction

There is a highly variable rate of decline in β-cell function after diagnosis of type 1 diabetes mellitus (T1DM); many patients retain detectable insulin secretion for years or decades (1). The assessment of β-cell function can help physicians to evaluate and manage T1DM patients. Several studies, including the Diabetes Control and Complications Trial (DCCT), have demonstrated that preservation of β-cell mass, in parallel with good glucose control, reduces the risk of serious hypoglycemia in the long term. Moreover, the higher the C-peptide levels, the lower the incidence of microvascular complications (MC), such as diabetic retinopathy, neuropathy, and diabetic renal disease (1–14).

Direct measurement of endogenous insulin secretion would be the most accurate method of evaluating β-cell function; however, it presents some limitations such as, low accuracy for very low levels of insulin, inability to differ between insulin and its intermediates, such as proinsulin, inability to differ between endogenous and exogenous insulin, and low reproducibility of the insulin dosage in peripheral blood by virtue of its first-pass hepatic extraction (15).

Thus, C-peptide measurements during the mixed-meal tolerance test (MMTT) have been recommended as gold standard to evaluate the degree of β-cell function. C-peptide is a byproduct of the enzymatic cleavage of proinsulin to insulin; it is co-secreted by pancreatic β-cells in equimolar concentrations with insulin (16–27). However, the time demand necessary to perform stimulation tests, as well as their usual unavailability, limits their use in clinical practice (28).

In the face of this caveat, insulin-dose adjusted A1c (IDAA1c) is an easy and fast alternative to evaluate pancreatic β-cell function. IDAA1c is a model of insulin-dose adjusted glycated hemoglobin A1c, calculated as “A1c (%) + 4x insulin dose (units per kilogram per 24 h) (28)”.

IDAA1c was first described in 2009 by the Hvidoere Study Group (28). The rationale behind the use of both total insulin dose and glycated hemoglobin in the same formula is to reduce the influence of the treatment regimen, since patients with different β-cell functions could present similar glycated hemoglobin values only by intensifying the treatment, which would limit the use of any of the two parameters separately. In that study, stimulated C-peptide, insulin dose (IU/kg), and A1c levels were periodically evaluated in 275 T1DM patients during the first year after diagnosis. A negative correlation among stimulated C-peptide, A1c, and insulin dose was demonstrated. Statistical analysis of regression coefficients showed a factor of ~4 between the coefficients of these parameters (A1c, 0.21; insulin dosage, 0.94), leading to the IDAA1c formulation as A1c (%) + 4x insulin dose. A linear correlation between IDAA1c and C-peptide over a continuum of stimulated C-peptide values, 6 and 12 months after diagnosis was observed. In addition, it was demonstrated that the IDAA1c threshold, nine, corresponds to a predicted level of 300 pmol/l for the corresponding peak-stimulated C-peptide (28).

In this study we aim to analyze the association between IDAA1c and MC in patients with T1DM as well as with hypoglycemia, in a representative Brazilian population of T1DM patients.

## Materials and Methods

### Study Design and Subjects

This study is based on analyses from a previous study, “microvascular complications in T1DM—a comparative analysis of patients treated with autologous non-myeloablative hematopoietic stem-cell transplantation (AHST) and conventional medical therapy.” The study design and data collection methods have been described elsewhere (29). Briefly, cross-sectional data of 24 T1DM patients, treated with AHST, were compared with those of 144 patients who received conventional therapy (CT) from the Brazilian Type 1 Diabetes Study (BrazDiab1).

**Figure 1:**
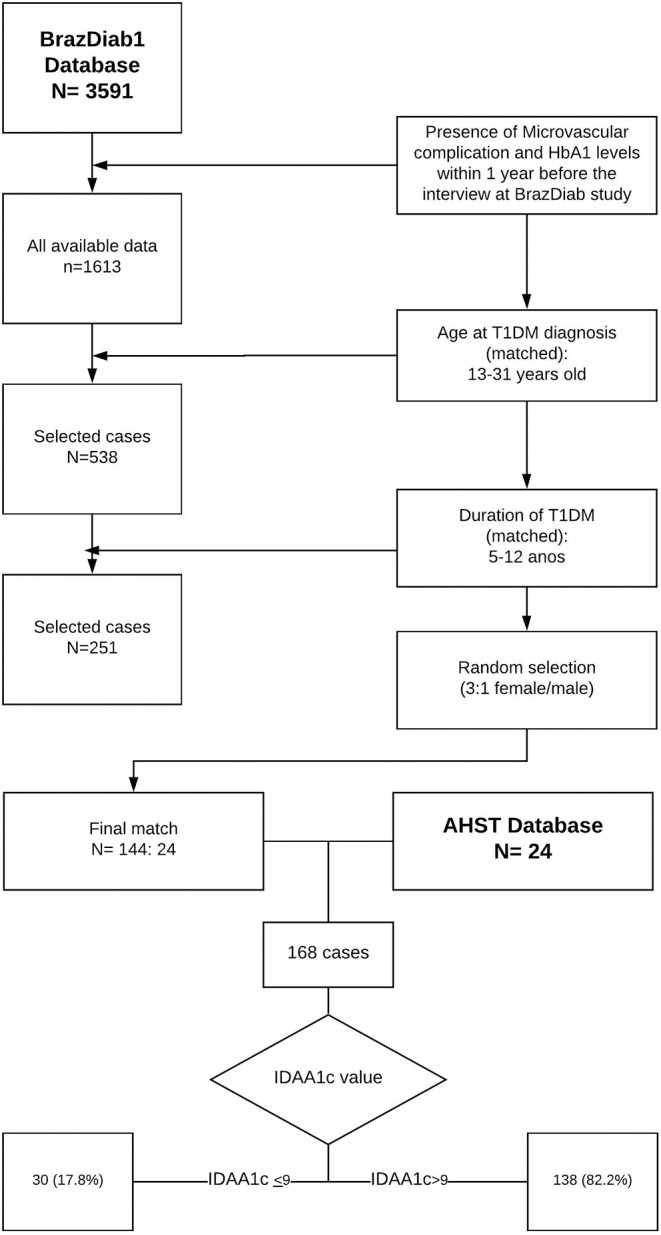
Case selection from AHST and CT group for data analysis.

The BrazDiab1 is the largest observational multicenter study in T1DM in Brazil. Assessed patients from 20 cities (population> 100,000) from all five Brazilian geographic regions (North, Northeast, Southeast, South, and Midwest). A total of 3,591 patients were enrolled in BrazDiab1, but only 1,613 had microvascular screening data available for this study. After AHST matching data (age at diagnosis of diabetes, duration of diabetes and gender) a total of 144 patients were included in the final analysis (Figure 1). All the 144 patients of CT were regularly followed by endocrinologists and were on insulin therapy as treatment for T1DM, with 87.5% of them using rapid acting and intermediate/long-acting insulin, 11.1% only intermediate/long-acting insulin and 1.4% were on insulin pump therapy (29, 30).

Complete study protocol of AHST was presented elsewhere (31). In brief, after peripheral hematopoietic stem cells mobilization with cyclophosphamide plus a granulocyte-colony stimulating factor, stem cells were harvested from the peripheral blood through apheresis. After mobilization, patients were conditioned with rabbit anti-thymoglobulin (ATG) plus cyclophosphamide. Posteriorly, intravenous infusion of autologous hematopoietic stem cells was performed with the aim of restoring immune balance.

The primary endpoint was to analyze the presence of MC, residual function of β-cell (through IDAA1c), insulin dose, and A1c in the well-matched groups.

In the present study, 168 patients of the initial study (144 from CT plus 24 from AHST) were re-subdivided into two groups, according to their IDAA1c values (IDAA1c ≤ 9 or IDAA1c > 9). Then, the prevalence of MC, hypoglycemia, and severe hypoglycemic events were compared between groups. Data on hypoglycemia were not presented in the original study (29).

### Microvascular Complications

Diabetic retinopathy was screened by fundoscopy by a local ophthalmologist; diabetic renal disease (DRD) was screened by measurements of albuminuria and glomerular filtration rate, estimated by the Cockcroft–Gault equation (32). Distal symmetric polyneuropathy was assessed by asking the patients about symptoms of neuropathy, including paresthesia, dulled sensation, and pain in the feet and using a neurological examination on the sensation of perception of vibration and 10-g monofilament pressure at the distal plantar of both great toes and metatarsal joints. The presence of MC was defined according to the American Diabetes Association Recommendations (33).

### Hypoglycemia

Hypoglycemia was assessed by asking the patient if he/she had any episode of hypoglycemia and/or severe hypoglycemia (SH) in the month prior to the interview, as well as the number of hypoglycemic episodes in the same period. Hypoglycemia was defined as capillary blood glucose level <60 mg/dL; severe hypoglycemia (SH), as an episode of hypoglycemia that required the assistance of another person to treat with oral carbohydrate or intravenous glucose due to altered consciousness or seizure (34).

### Statistical Analysis

Continuous variables were presented as mean and SD, and the categorical variables as absolute frequency and percentage. The comparative analysis between the two groups was estimated using the unpaired Student *t*-test, or Mann–Whitney *U*-test for continuous variables and the Pearson's chi-square test, Likelihood ratio test or Fisher's exact test for the categorical variables. Likelihood ratio test and Fisher's exact test was used to compare the MC of the analysis. The Pearson's chi-square, Mann–Whitney and Fisher test were performed for comparison of hypoglycemic events. Results were considered significant at *p* < 0.05.

## Results

### Patient Characteristics

The patient data selection was showed on Figure 1. Of the 168 patients, 30 (17.8%) and 138 (82.2%) had IDAA1c ≤ 9 and IDAA1c > 9, respectively. The proportion of males was 56.7 and 73.2% in the IDAA1c ≤ 9 and IDAA1c > 9 groups, respectively (*p* = 0.073). During the data collection, the mean duration of diabetes was 8.8 years in the IDAA1c ≤ 9 group and 8.2 years in the IDAA1c > 9 group (*p* = 0.214). The age at diabetes diagnosis varied from 13 to 31 years; the mean was 19.6 years in the IDAA1c ≤ 9 group and 18.2 years in the IDAA1c > 9 group (*p* = 0.159). Demographic characteristics of the study population are summarized in Table 1.

**Table 1:** Gender, duration of T1DM and age distribution for the IDAA1c ≤ 9 and IDAA1c > 9 groups.

**Patient characteristics**	**IDAA1c≤9 group (*n* = 30)**	**IDAA1c>9 group (*n* = 138)**	***p*-value**
Male gender, *n* (%)	17 (56.7)	101 (73.2)	0.073^a^
Duration of diabetes, Years#	8.8 ± 1.88	8.23 ± 2.34	0.214^b^
Age at diagnosis, Years#	19.67 ± 4.94	18.29 ± 4,81	0.159^b^

a Q^2^ Fisher Test;

b *T Test; #mean ± SD*.

### Microvascular Complications

The prevalence of MC (diabetic renal disease, neuropathy, or retinopathy) was lower in the IDAA1c ≤ 9 group. Whereas, only 6.6% (2/30) of patients in this group developed any form of MC, 21.0% (29/138) of the IDAA1c > 9 group had at least one of them (*p* = 0.044). In IDAA1c ≤ 9 both patients with MC had diabetic renal disease, one had microalbuminuria and the other one had macroalbuminuria, either treated with CT. When the individual components of MC were assessed separately, the number of cases was larger in IDAA1c > 9 group, but the difference was not statistically significant (Table 2).

**Table 2:** Comparison of occurrence of diabetes microvascular complications in the IDAA1c ≤ 9 and IDAA1c > 9 groups.

**Patient characteristics**	**IDAA1c≤9 group (*n* = 30) !**	**IDAA1c>9 group (*n* = 138)**	***p*-value**
Any microvascular complication	6.6% (2/30)	21.0% (29/138)	0.044^c^
Diabetic renal disease	6.6% (2/30)	13.0% (18/138)	0.067^d^
Microalbuminuria	1	15	
Macroalbuminuria	1	3	
Diabetic neuropathy	0	6.5% (9/138)	0.365^d^
Diabetic retinopathy	0	5.7% (8/138)	0.353^d^

*! IDAA1c ≤ 9 group N = 30 (18 AHST +12 BrazDiab1)*.

c Likelihood ratio test;

d *Q^2^ Fisher Test*.

### Hypoglycemic Events

The proportion of patients who reported at least one episode of hypoglycemia in the last month was lesser in the IDAA1c ≤ 9 group (43.3%) than in the IDAA1c > 9 group (64.7%) (*p* = 0.030); however, the rate of hypoglycemic episodes per patient per month was similar between groups (3.1/patient/month vs. 2.9/patient/month; *p* = 0.088). Regarding severe hypoglycemia, the proportion of patients presenting at least one episode in the last month and the rate of episode/patient/month were similar between groups (6.7 vs. 13.2%; *p* = 0.535; and 0.1/patient/month vs. 0.25/patient/month; *p* = 0.321). In the IDAA1c > 9 group, there were no data on the presence of hypoglycemia in two patients and no data on severe hypoglycemia in eight patients (Table 3).

**Table 3:** Comparison of Hypoglycemic events in the IDAA1C ≤ 9 and IDAA1c > 9 groups.

**Patient characteristics**	**IDAA1c≤9 group (*n* = 30)!**	**IDAA1c>9 group (*n* = 138)**	***p*-value**
Hypoglycemia event, at least 1 episode in the last month	43.3% (13/30)	64.7% (88/136)^e^	0.030^g^
Hypoglycemia per patient, any event in the last month	3.1 (93/30)	2.9 (407/136)^e^	0.088^h^
Severe hypoglycemia, at least 1 episode in the last month	6.7% (2/30)	13.2% (18/136)^f^	0.535^i^
Severe hypoglycemia per patient, any event in the last month	0.1/patient (3/30)	0.25/patient (33/130)^f^	0.321^h^

! IDAA1c ≤ 9 group N = 30 (18 AHST +12 BrazDiab1);

e 2 patients did not report hypoglycemic events;

f 8 patients did not report hypoglycemic events;

g Pearson

h Mann-Whitney;

i *Fisher*.

## Discussion

In this study, we analyzed the association between IDAA1c levels and hypoglycemia and MC. We found that in the group of patients with IDAA1c ≤ 9, there was lower prevalence of MC, as well as fewer episodes of hypoglycemia, in the month prior to the analysis.

Since its first description in 2009, the relationship between IDAA1c and C-peptide levels have been studied severally, increasing the credibility of this parameter as a marker of β-cell function (35–38). In 2014, the same group replicated its early findings in a different population. Assessing 129 Danish children and adolescents with new-onset T1DM, they have confirmed the relationship between IDAA1c ≤ 9 and peak-stimulated C-peptide levels >300 pmol/L; this shows a linear relation between these two variables at 6 and 12 months after diagnosis (35).

Another trial that included an adult population (67 individuals aged 7–45 years) with 24 months of follow-up, confirmed the good specificity of IDAA1c in predicting C-peptide levels. In 99% of cases, when IDAA1c was <9, peak C-peptide levels were >200 pmol/L (34). However, it is important to emphasize that in both studies, the sensitivity of IDAA1c was shown to be lower than specificity, with some individuals presenting peak-stimulated C-peptide levels >200–300 pmol/L, even when IDAA1c was >9 (35, 36).

Recently, a new formula to estimate residual beta-cell function in children was published. In addition to HbA1c, and insulin dose, age, gender and BMI were included. The predictive value for 90-min stimulated C-peptide was significantly higher than IDAA1c. However, this clinical model was evaluated only in a small number of children with recent-onset type 1 diabetes (37).

In addition to its correlation with C-peptide levels, other studies have indirectly evaluated the relationship between IDAA1c and β-cell function. A periodic measurement of IDAA1c during 6 years in a large longitudinal observational study of 3,657 children and adolescents with newly diagnosed type 1 diabetes showed that older age at onset of diabetes, absence of Diabetic ketoacidosis (DKA) and initial autoantibody negativity were associated with a higher possibility of patients presenting values of IDAA1c ≤ 9 at any moment during the 6 years of study (38). On the other hand, positive autoantibodies at T1DM onset are predictors for a shorter period of IDAA1c values ≤ 9 during the observational study period (38). Previously, we have applied IDAA1c to evaluate and compare β-cell function in patients with newly diagnosed T1DM treated with AHST and patients who received conventional therapy from the Brazilian Type 1 Diabetes Study Group (BrazDiab1). We demonstrated that after a median of 8 years of diagnosis, the proportion of individuals with IDAA1c ≤ 9 was about 10-fold higher in the first group (29). Similarly, Type 1 NEw ONset Study (NeOn) that evaluated 1,048 patients <19 years old, showed that the number of DKA episodes increased in parallel with IDAA1c levels. While the proportion of participants with IDAA1c ≤ 9 decreased from 23% at 12 months to 7% at 36 months, the rate of patients developing diabetic ketoacidosis (DKA) increased from 1% in the first year after diagnosis to 6% in years 2 and 3 (39). It is known that both advanced age at the onset of diabetes and the absence of DKA correlate with better residual β-cell function (40–46), whereas the presence of serum autoantibodies, as well as DKA at T1DM onset, may be related to an aggressive autoimmune reaction and accelerated destruction of beta cells (46, 47).

On the other hand, numerous studies show that the preservation of β-cell function results in fewer MC (1–15), where the DCCT provided the strongest evidence. In DCCT, C-peptide responders (C-peptide response above 0.2 nmol/l) had significantly lower rates of albumin excretion (*P* = 0.027) and nearly significantly lower prevalence of retinopathy (*P* = 0.057) than non-responders, after adjusting for age and sex (12, 15). Thus, since IDAA1c ≤ 9 has been associated with better pancreatic β-cell reserve, and this condition reflects in lower MC, this may justify our finding of less prevalence of MC and hypoglycemia in the IDAA1c ≤ 9 group.

Data from literature show a reduced risk of hypoglycemia in patients with better residual β-cell function (48, 49). In our study, 43.3% of patients in the IDAA1c ≤ 9 group presented with at least 1 hypoglycemic episode in the month prior to the analysis and 63.7% in the IDAA1c > 9 group (*p* = 0.03). This is in accordance with the DCCT trial that showed reduced incidence of hypoglycemia in individuals with better β-cell function (48).

A recent study that included patients undergoing immunomodulatory therapy, showed that a 4-h area under the curve (AUC) of C-peptide levels is a robust predictor of number of hypoglycemic events and demonstrated an inverse correlation between a continuous scale of IDAA1c values and the 4-h AUC of C-peptide. However, there was no difference in hypoglycemic rates between IDAA1c ≤ 9 and IDAA1c > 9 groups in our study. Probably, the arbitrary categorization of two groups simply based on the threshold, nine, would not be appropriated to this population. Recent reports indicate that reduced rates of hypoglycemia are observed across a range of C-peptide values with no threshold or breakpoint (50).

By evaluating episodes of severe hypoglycemia in our study, the proportion of patients with at least one episode, and the rate of episodes of severe hypoglycemia per patient were similar between groups. In line with our findings, the NEON study showed no significant difference in IDAA1c values between those participants with or without severe hypoglycemia. Whereas, the percentage of participants with IDAA1c ≤ 9 decreased from 23% at 12 months to 7% at 36 months, the rate of patients reporting severe hypoglycemic episodes only increased from 2 to 4% in the same period, but without statistical significance (51). Another study that included data from 3,320 Danish patients aged 0–18 years with mean diabetes duration of 4 years showed similar results. Although initially IDAA1c ≤ 9 had been correlated with fewer episodes of severe hypoglycemia, the difference was no longer significant in the final adjusted model (52).

In this study, we showed a relationship between IDAA1c and MC in T1DM patients. However, its retrospective nature, cross-sectional design, population size, and the use of self-reported assessment of hypoglycemic episodes (which could lead to memory bias), are recognized limitations. Considering that continuous glucose monitoring levels were not evaluated, silent episodes would not be accounted. On the other hand, strengths were the evaluation of IDAA1c in a population with long term disease and the inclusion of a large number of patients undergoing immunomodulatory therapy, which allowed us to evaluate a significant proportion of patients presenting IDAA1c ≤ 9, even after a long disease duration. Therefore, this simple parameter of estimation of β-cell function may be useful in defining patients with T1DM who deserve greater attention in the sense of prevention-associated complications.

In conclusion, patients with T1DM with IDAA1c ≤ 9 presented a lower frequency of MC, as well as hypoglycemic episodes. Thus, the use of IDAA1c may be a very simple method to help physicians in the prediction of patients with higher risk of these complications and in the management of patients with T1DM. However, since most patients in the IDAA1C ≤ 9 group (18 of 30) were treated with AHST, these results may not be widespread for general T1DM population with long duration of diabetes. Thus, further studies with a greater number of patients, who are followed for a longer period of time, are necessary for confirmation of these findings and determine the clinical application of IDAA1c.

## Data Availability Statement

The datasets generated for this study are available on request to the corresponding author.

## Ethics Statement

The studies involving human participants were reviewed and approved by the University of Ceara Hospital Research Ethics Board (Protocol no. 1886743). The patients/participants provided their written informed consent to participate in this study.

## Author Contributions

JP-S contributed to the analysis of data and writing of the manuscript. CC, KM, DM, JD, MO, and MG contributed with the analysis of data. RM, LZ, LB, AM, VF, AH, and CN contributed to the writing, reviewing, and editing of the manuscript.

### Conflict of Interest

The authors declare that the research was conducted in the absence of any commercial or financial relationships that could be construed as a potential conflict of interest.
